# Monitoring the Season–Prevalence Relationship of *Vairimorpha ceranae* in Honey Bees (*Apis mellifera*) over One Year and the Primary Assessment of Probiotic Treatment in Taichung, Taiwan

**DOI:** 10.3390/insects15030204

**Published:** 2024-03-19

**Authors:** Yi-Hsuan Li, Yu-Hsin Chen, Fang-Min Chang, Ming-Cheng Wu, Yu-Shin Nai

**Affiliations:** 1 Doctoral Program in Microbial Genomics, National Chung Hsing University and Academia Sinica, Taichung 402202, Taiwan; lisa19980923@gmail.com; 2 Department of Entomology, National Chung Hsing University, Taichung 402202, Taiwan; yhchen1806@gmail.com (Y.-H.C.); a0930256530@gmail.com (F.-M.C.)

**Keywords:** *Vairimorpha ceranae*, *Apis mellifera*, probiotic treatment, *Leuconostoc mesenteroides*, microsporidiosis

## Abstract

**Simple Summary:**

The *Vairimorpha ceranae* prevalence and the temperature were surveyed and recorded at the National Chung Hsing University apiary for one year. A positive correlation was found between *V. ceranae* and temperature, suggesting that the prevalence or activity of *V. ceranae* increases as temperatures rise. Furthermore, the anti-microsporidia activity of the lactic acid bacteria *Leuconostoc mesenteroides* TBE-8 against *V. ceranae* was assessed for the management of microsporidiosis.

**Abstract:**

Microsporidiosis, which is caused by the pathogen *Vairimorpha ceranae*, is a prevalent disease in the honey bee (*Apis mellifera*) and might lead to significant adult honey bee mortality. In this study, we conducted an annual survey of the mature spore load of *V. ceranae* in the guts of nurse bees and forager bees in the apiary of National Chung Hsing University (NCHU) in Taiwan. The results indicated that, on average, honey bees hosted approximately 2.13 × 10^6^ mature spore counts (MSCs)/bee in their guts throughout the entire year. The highest number of MSCs was 6.28 × 10^6^ MSCs/bee, which occurred in April 2020, and the lowest number of MSCs was 5.08 × 10^5^ MSCs/bee, which occurred in November 2020. Furthermore, the guts of forager bees had significantly higher (>58%) MSCs than those of nurse bees. To evaluate the potential of the probiotic to treat microsporidiosis, the lactic acid bacterium *Leuconostoc mesenteroides* TBE-8 was applied to honey bee colonies. A significant reduction (>53%) in MSCs following probiotic treatment was observed, indicating the potential of probiotic treatment for managing microsporidiosis. This research provided information on *V. ceranae* MSCs in the honey bee gut at NCHU in Taiwan and the MSCs’ correlation with the annual season. Furthermore, a potential probiotic treatment for microsporidiosis was assessed for future management.

## 1. Introduction

The honey bee (*Apis mellifera*) plays a crucial role as a primary pollinator in crop production, contributing significantly to the agricultural economy. By 2020, honey bees had contributed approximately USD 6.4 billion to crop yield in the USA, showcasing their immense economic value and extensive benefits for global agriculture [1,2,3,4]. Despite their pivotal role, honey bee colonies face various stressors, including pesticide use, environmental pollution, food scarcity, inadequate nutrition, and multiple diseases, ultimately leading to colony decline [2,5]. The health of honey bee colonies is jeopardized by a range of pathogens, including bacteria, viruses, and fungi, resulting in diseases such as American foulbrood (caused by *Paenibacillus larvae*) [5], chalkbrood disease (caused by *Ascosphaera apis*) [6], and microsporidiosis (caused by *Vairimorpha* spp.). Microsporidiosis, a disease specific to *A. mellifera*, has been reported in honey bee populations worldwide [7,8,9,10]. The causal agents are *Vairimorpha ceranae* and *Vairimorpha apis*, both of which are fungal pathogens. The extensive global trade among beekeepers and honey bee pollination have significantly contributed to the high prevalence and abundance of *V. ceranae* and *V. apis* in bee guts [11,12,13].

Regarding the host range, *V. ceranae* was initially discovered in the Eastern honey bee (*Apis cerana*) in 1996 [14]. *V. ceranae* was later identified as a natural infection in *A. mellifera* colonies in 2005 [15,16]. Over time, the range and prevalence of *V. ceranae* have significantly expanded, and *V. ceranae* has become the predominant species in numerous regions, such as Spain, Italy, Israel, Greece, and Turkey [17]. Epidemiological evidence suggests that *V. apis* was replaced by *V. ceranae* [9]. Despite the widespread dominance of *V. ceranae* in *A. mellifera* colonies, its prevalence demonstrates a seasonal infection pattern that varies across geographical areas. Some areas, such as Serbia, Germany, and New Zealand, showed a higher prevalence in spring. However, other areas, such as Uruguay, exhibited a higher incidence from the winter to the spring [12,18,19]. A preliminary study revealed that *V. ceranae* has higher loads in the spring and fall and that the intensity of *V. ceranae* is negatively correlated with low temperature; this phenomenon relating the intensity of *V. ceranae* and temperature has been described in studies in Taiwan [12].

*Vairimorpha* sp. is an obligate intracellular fungus that is transmitted to hosts through mature spores [2,20]. Throughout all developmental stages of *Vairimorpha* sp., diplokaryotic nuclei are present, and a long flexible polar tube appears in the mature spores [21]. *Vairimorpha* sp. spores germinate in the midgut lumen of bees, extrude polar filaments, and transfer sporoplasm into epithelial cells [17]. The sporoplasm develops into a meront during the merogonic phase, and then matures into spores. These environmental spores are released into the midgut lumen through cell lysis, initiating the reproductive cycle, or are expelled via defecation as free spores [2,17]. The proliferation of *V. ceranae* causes lesions in the bee gut and may decrease the digestive ability of the bee, which causes further nutritional and energetic stress [17]. Infection by *V. ceranae* weakens *A. mellifera*, and severe cases can result in bee mortality, which is often correlated with significant colony losses. These infections are also associated with various physiological impairments, contributing to reduced honey production, malnutrition, shortened lifespans, and increased mortality among adult honey bees [2,22].

The exploration of treatments for microsporidiosis is crucial. Although the chemical fumagillin has been widely utilized for several decades for the treatment of microsporidiosis in honey bees, recent studies suggest its potential ineffectiveness against *V. ceranae* infections and even suggest that fumagillin may exacerbate the severity of microsporidiosis [2,23,24]. Several extracts and natural supplements, such as *Agaricus blazei* mushroom extract, *Andrographis paniculate* decoction, and BEEWELL AminoPlus, have been extensively researched. These treatments have shown promising results in enhancing bee survival and reducing spore loads following oral administration, providing new options for managing *V. ceranae* infections [24,25,26].

Additionally, several studies have investigated the use of probiotics, such as those in the *Lactobacillus*, *Bacillus*, *Bifidobacterium*, *Enterococcus*, and *Pediococcus* genera, for controlling microsporidiosis in honey bees [27]. These probiotics have demonstrated the ability to enhance honey bee survival, reduce *Vairimorpha* spores, increase adult bee populations, and boost honey production [28,29,30].

As mentioned above, a seasonal increase in *V. ceranae* infection intensity was observed in Taiwan, with peak pathogen loads reported during spring and fall, suggesting that *V. ceranae* is suitable for subtropical to tropical climates [12]. However, these data included average temperatures between 15 °C and 30 °C, which were lower than the temperature recorded in Taichung. Previous research has indicated a negative correlation between pathogen load and temperature for *V. ceranae*; a temperature of 15 °C is associated with the highest *V. ceranae* spore count recorded, suggesting an association between microsporidiosis and temperature [12]. In this study, we conducted a survey of colonies within the apiary at National Chung Hsing University, Taichung, Taiwan. We collected *V. ceranae* samples from the apiary and recorded spore counts to assess the pathogen load of *V. ceranae* in colonies and to determine the correlation between infection intensity across different seasons and temperatures. Additionally, we conducted a preliminary evaluation of the potential effects of the honey bee probiotic, *Leuconostoc mesenteroides* TBE-8. In this study, the effectiveness of probiotics in controlling *V. ceranae* was discussed, and this information provided new insights for the treatment of microsporidiosis.

## 2. Materials and Methods

### 2.1. Honey Bees

Honey bee adults were collected from honey bee (*A. mellifera*) colonies via the NCHU apiary (National Chung Hsing University, Taichung, Taiwan). Worker bees were collected weekly from 4–6 colonies throughout the year (for a total of forty-nine weeks) from April 2020 to March 2021. A total of five nurse bees or forager bees were collected as one sample. The temperature of the apiary was recorded weekly. For the probiotic treatment experiment, two colonies were used to be the treatment group, and another two colonies were used to be the control group. Each colony contained a young, normal, egg-laying queen and had a working population of 7–8 frames of combs with larvae, pupae, honey, and pollen.

### 2.2. Mature Spore Counts

The collected honey bees were anesthetized at −20 °C for midgut tissue dissection. Five midgut tissues were pooled in 500 μL of 1× TE buffer (0.1 M Tris, 0.01 M EDTA, pH 9.0) in 1.7 mL microtubes as one sample and homogenized. The number of mature spores was observed and calculated by using a hemocytometer (Paul Marienfeld GmbH & Co. KG, Lauda-Königshofen, Germany) under 400× light microscopy (WHITED).

### 2.3. Treatment of Probiotics

For the probiotic treatment, two colonies were treated, and two colonies were used as the control group for five weeks from September to October. The probiotic *Leuconostoc mesenteroides* TBE-8 bacterial powder was prepared via the lyophilization of a 2 L culture. The viable bacterial count indicated that 1 g of the TBE-8 bacterial powder contained 3 × 10^12^ colony-forming units (CFUs). The treatment group honey bee colonies were provided 500 mL of 50% sucrose syrup containing 1 g of the TBE-8 bacterial powder two times a week (on Monday and Thursday), and the control group colonies were provided with 50% sucrose syrup. The probiotic treatment started on 28 September 2020 and continued for one month until 22 October 2020, as this period precedes the anticipated increase in *V. ceranae* and is suitable for therapeutic evaluation. A total of four bee hives were subjected to the experiment; two bee hives were treated with probiotics (treatment group), and another two bee hives were fed with same food and quantity without probiotics (control group). The monitoring of *V. ceranae* infection was performed by detecting MSCs and genome copies from September 2020 to December 2020 (early autumn to winter), which were considered before, during, and after probiotic treatment.

### 2.4. Statistical Analysis

Statistical analysis and plotting were performed with R 4.0.3, and the R packages included ggplot2, ggpubr, and ggpmisc [31,32,33,34]. The statistical analysis of probiotic-detected bar plots was performed using the R package rstatix [35].

## 3. Results

### 3.1. Season–Prevalence Relationship of V. ceranae

**Figure 1:**
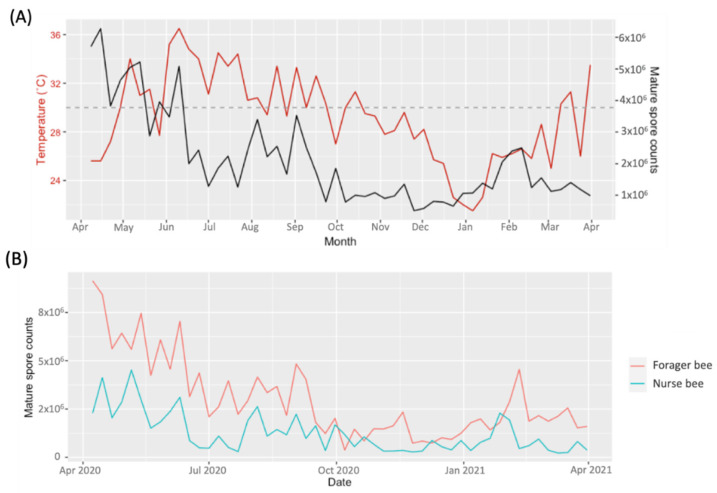
From April 2020 to April 2021, the *Vairimorpha ceranae* mature spore count was measured. (**A**) Record of spore counts and temperature (°C) and the dotted line indicates 30 °C. (**B**) Mature spore count record of nurse and forager bees.

The annual records of the MSC in the NCHU apiary are depicted in Figure 1. The results revealed the continuous presence of MSCs throughout the entire year. The highest peaks in MSC (ranging from 2 × 10^6^~6.28 × 10^6^ MSC/honey bee) were noted from April to June 2020, followed by August to September 2020, ranging from 7.9 × 10^5^~3.53 × 10^6^ MSC/honey bee. Another peak was observed in February 2021, at 2.1 × 10^6^~2.5 × 10^6^ MSCs/bee (Figure 1). Based on our observations, temperatures increased from April (27.2 °C) to July (34.4 °C), while MSC decreased from 6.28 × 10^6^ to 1.26 × 10^6^ MSC/honey bee (Figure 1A). It was further observed that the MSC consistently decreased when the apiary temperature was at or above 30 °C from June to November 2020. However, the MSC increased as the temperature decreased below 30 °C from December 2020 to February 2021 (Figure 1A). Additionally, on average, the detection of MSCs was higher in forager bees than in nurse bees from April 2020 to October 2020 and from January 2021 to March 2021 (Figure 1B).

**Figure 2:**
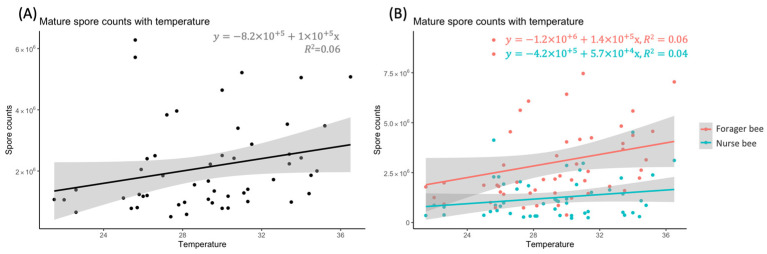
Mature *Vairimorpha ceranae* spore counts with temperature correlations from April 2020 to April 2021. (**A**) Scatter plots of worker bee temperature; (**B**) scatter plots of nurse bee and forager bee temperature; *n* = 52; gray area = 95% confidence limit.

An analysis of the correlation between temperature and MSC revealed positive correlations between MSC and temperature (Figure 2). The highest MSC was 6.28 × 10^6^ in April 2020, and the lowest MSC was 5.08 × 10^5^ in November 2020 (Figure 2A). This positive correlation was also found for both nurse bees and forager bees (Figure 2B). For the nurse bees, the highest MSC was 4.5 × 10^6^ in May 2020, the lowest MSC was 3 × 10^5^ in July 2020, the difference between the highest and lowest MSC for nurse bees was 4.2 × 10^6^. For the forager bees, the highest MSC was 9.1 × 10^6^ in April 2020, the lowest MSC was 3.8 × 10^5^ in October 2020, and the difference between the highest and lowest MSC was 8.7 × 10^6^ MSC (Figure 2B). Therefore, the difference was more noticeable in forager bees than in nurse bees, and the temperature difference might be a factor that contributes to the MSC in honey bees.

### 3.2. Efficacy of Probiotic Treatment

**Figure 3:**
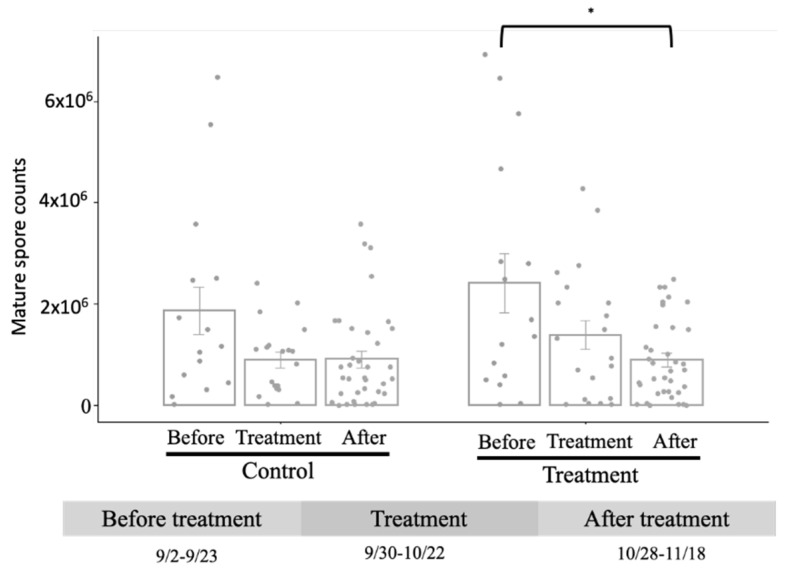
Detection of MSC from *V. ceranae* following probiotic treatment. Significant differences before and after probiotic treatment were determined using one-way ANOVA with a Bonferroni correction; * *p* value < 0.05; error bar = SE; *n* = 52.

For the probiotic treatment of the colonies, in the *L. mesenteroides* treatment group, the average MSC before treatment was 2.4 × 10^6^. After *L. mesenteroides* treatment, the MSC experienced a significant decrease by 53.6% from the average load before treatment (*p* value < 0.05), from 1.38 × 10^6^ (treatment) to 8.86 × 10^5^ (after treatment). From the observations, the highest MSC loads before and after treatment were 1.2 × 10^7^ and 8.3 × 10^6^, respectively, which decreased by 30%. Though the average MSC after treatment (9.01 × 10^5^) was lower than that before treatment (1.87 × 10^6^), there was no significant difference between before and after treatment in the control group. Furthermore, the MSC average load increased from 8.84 × 10^5^ (before treatment) to 9.01 × 10^5^ (after treatment) in the control group, suggesting that *L. mesenteroides* has anti-mature spore activity to *V. ceranae* (Figure 3).

## 4. Discussion

The presence of *V. ceranae* in *A. mellifera* colonies in the NCHU apiary, Taichung city, demonstrated fluctuations in MSC throughout the season. The year-round presence of *V. ceranae* has been reported, with variations in peak spore loads across different climates. A report from 2008–2009 showed a negative correlation between temperature and *V. ceranae* load; the highest load was observed in the winter, with an MSC of approximately 7 × 10^6^ at 15 °C to 20 °C [12,17]. Our survey revealed that MSC increased as temperature increased, and the highest MSC load we recorded was higher than that in a previous study from 2008 to 2009, which was different from the findings of Chen et al. (2012). Notably, the temperature range recorded in 2008–2009 was 15 to 30 °C, which was lower than our observations from 2020 to 2021 in Taichung (21.5 to 36.5 °C) [12]. This finding suggested that high temperatures led to lower MSCs in our study, indicating that global warming might adversely affect the prevalence of *V. ceranae*. Consequently, the temperature we recorded during 2020–2021 was higher than that recorded during 2008–2009. Additionally, our data showed different MSC trends than those in the 2008–2009 report, suggesting that temperature caused the different incidences of *V. ceranae* infection and MSC throughout the year.

Increased MSCs were also observed in forager bees within this study. On average, the MSC in forager bees was 3 × 10^6^, while MSC in nurse bees was 1.2 × 10^6^, and the forager MSC was approximately 58% higher than the nurse bee MSC. Discrepancies in *V. ceranae* spore loads between nurse bees and forager bees have been documented in earlier research [8,36,37]. Positive correlations between temperature and MSC were evident for forager bees but were less noticeable for nurse bees during the autumn to spring period, suggesting that forager bees might obtain mature spores while collecting nectar and pollen, particularly during the spring season. *V. ceranae* does not have enough time to develop during the nurse bee stage. Because *V. ceranae* growth extends from the nurse bee stage, *V. ceranae* in forager bees has more time to develop, which is a potential reason for the higher MSC in forager bees [8].

A decrease in the MSCs was observed after probiotic treatment, indicating that the effectiveness of the anti-*V. ceranae* treatment was specific to the mature spore stage in the intracellular phase. According to the genome copy number (GCN) (Supplementary Figure S1), probiotic treatment had a more significant impact on MSC, suggesting that probiotic treatment might influence the mature spore stage of *V. ceranae* rather than the merogony stage. The intracellular life cycle of *V. ceranae* includes the proliferative phase (merogony) and the sporogonic phase (sporogony). After infection, the microsporidia were first replicated at the intracellular stage, followed by the formation and release of mature spores from host cells [38]. Upon returning to the environment, there is an opportunity to infect new bees [38]. Therefore, the suppression of the mature spore load within the bee colony can reduce the opportunities of MSCs in the environment, thereby reducing the probability of new emergency honey bees suffering microsporidia infection. This may lead to an overall reduction in the *V. ceranae* load within the colony. Based on the outcomes of this study, probiotic treatment might reduce the mature spore load of *V. ceranae* within the bee colony. In the future, a large-scale experiment is needed to further elucidate the efficacy of probiotic treatment on honey bee microsporidiosis.

## 5. Conclusions

This one-year surveillance of *V. ceranae* incidence in *A. mellifera* apiaries at NCHU in Taiwan revealed noteworthy correlations between MSC incidence and environmental factors and the variation in spore loads among different social castes of honey bees. Our data revealed a positive correlation between MSC incidence and temperature, suggesting that temperature differences might impact the development of *V. ceranae*. However, further experiments are needed to validate these associations. A comparison of the MSCs between nurses and forager bees revealed distinct loads among the different life stages, indicating that the development of MSC loads follows the infection process in various honey bee life stages. Preliminary data on probiotic treatment exhibited efficacy in controlling MSC, suggesting the potential to prevent honey bees from reaching the mature spore stage of *V. ceranae* infection. Our study delineated the intricate relationship between environmental factors and *V. ceranae* across different social castes while introducing probiotics as a potential treatment for microsporidiosis control. Future research endeavors should focus on developing a comprehensive microsporidiosis treatment process in apiculture.

## Data Availability

The data presented in this study are available upon request from the corresponding authors.

## Supplementary Materials

The following supporting information can be downloaded at: https://www.mdpi.com/article/10.3390/insects15030204/s1, Figure S1: Total spore counts and genome copy numbers of the control group (A and C) and treatment group (B and D) from September to December.
