# Supplementary material for: Monitoring the Season–Prevalence Relationship of Vairimorpha ceranae in Honey Bees (Apis mellifera) over One Year and the Primary Assessment of Probiotic Treatment in Taichung, Taiwan
Source: Insects. 2024 Mar 19;15(3):204. doi: 10.3390/insects15030204 (PMC10970892; doi:10.3390/insects15030204)
Supplement: Supplementary file 1 [file insects-15-00204-s001.zip › 20240211-supp. meterial.pdf]

Monitoring the season-prevalence relationship of *Vairimorpha ceranae* in honey bees (*Apis mellifera*) over one year and primary assessment of probiotic treatment

Yi-Hsuan Li[1], Yu-Shin Chen[2], Fang-Min Chang[2], Ming-Cheng Wu[1, 2]*, Yu-Shin Nai[1, 2]*

[1] Doctoral Program in Microbial Genomics, National Chung Hsing University and Academia Sinica, Taiwan.

[2] Department of Entomology, National Chung Hsing University, Taichung, Taiwan.

*Correspondence: Ming-Cheng Wu: mcwu@nchu.edu.tw; Yu-Shin Nai: ysnai@nchu.edu.tw

Supplementary Materials

Supplementary Methods

DNA extraction

Total 100 μL of the homogenized suspension was used was for DNA extraction by EDNA HISPEX (Easy DNA High-Speed Extraction) Tissue Kit (Fisher Biotec, Australia). Briefly, 64 μL of solution 1A and 16 μL of solution 1B was mixed with 100 μL homogenized suspension, incubated at 95 °C for 20 minutes, then mixed with

20 μL solution 2 for total 200 μL. The DNA sample was then 100-fold diluted with sterilized ddH_2O and store at −20 °C until used.

Absolutely quantitation of *V. ceranae* genome copy number

For the absolutely quantitation, a standard curve based on the serial-dilution of Nc-MSP was established as followed; Centrifuged at 16000 ×*g* for 3 min to removed midgut tissue of honey bees and the 1×10^7 spores per mL were harvest from the homogenized suspension as described above. The DNA was extracted as described above and then 10-fold serial dilution was performed for seven times to prepare the DNA sample with the spore concentrations from 1×10^6 spore / mL to 1×10^0 per mL. These DNA samples were subjected to real-time quantitative PCR (qPCR) to generate the standard curve of *V. ceranae* genome (y= -3.3043x + 37.909 R^2 = 0.9894). For qPCR, the ribosomal RNA gene specific primer set was design: SSU-F: 5'- CCCTGACTGGACGAACAGAAG-3'; SSU-R: 5'-CCAGCTTACGTCCTTGTTCAA A-3' was used to quantify the genome copy number of *V. ceranae* in both DNA of collected samples and DNA for standard curve. The linear standard equation for *V. ceranae* quantification was generated by plotting the crossing point (Cp) versus the $\log_{10}$ of the initial plasmid copy number as follows: y =-3.3043x+ 37.909, R^2 = 0.99. Real-time qPCR was performed by using iQ™

SYBR® Green Supermix (Bio-Rad) in a CFX Connect Real-Time PCR Detection System (Bio-Rad). Total reaction volume of 20 μL was consisted of 10 μL 2× iQ™ SYBR® Green Supermix (Bio-Rad), 1 μL per forward (10 mM) and reverse primers (10 mM) and 1 μL DNA template. The qRT-PCR program was performed as follows: 95 °C for 3 min, 40 cycle at 95 °C for 10 s, 59 °C for 30 s All samples were performed in five repeats. Convert the qPCR result to the spore number by formula: copy number $\times 2 \times 100 \times 100$. 2 was back calculation for the dilution of DNA extraction, one of 100 was for the diluted after DNA extraction and other was used 1 μL 100× DNA for qPCR.

Supplementary Figure 1

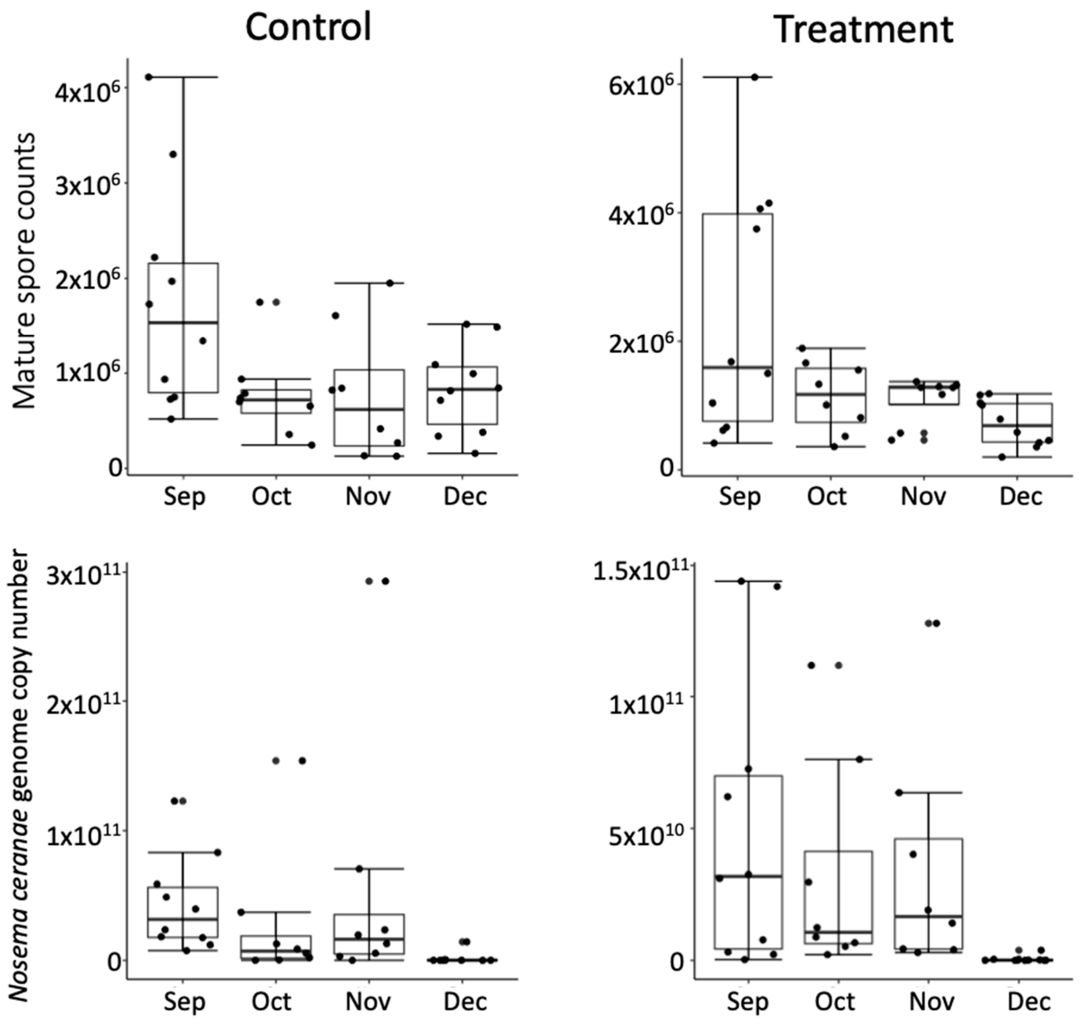

Figure S1. Total spore counts and genome copy numbers of the control group (A and C) and treatment group (B and D) from September to December.
